# Physical prehabilitation improves the postoperative outcome of associating liver partition and portal vein ligation for staged hepatectomy in experimental model

**DOI:** 10.1038/s41598-022-23744-2

**Published:** 2022-11-14

**Authors:** Noemi Daradics, Klara Levay, Ildiko Horvath, Noemi Kovacs, Domokos Mathe, Krisztian Szigeti, Attila Szijarto, Andras Fulop

**Affiliations:** 1 Department of Surgery, Transplantation and Gastroenterology, Hepato-Pancreatico-Biliary Surgical Research Center, Semmelweis University, Ulloi Street 78, 1082 Budapest, Hungary; 2 Department of Biophysics and Radiation Biology, Semmelweis University, Budapest, Hungary; 3 In Vivo Imaging Core Facility, Hungarian Centre of Excellence for Molecular Medicine, Szeged, Hungary

**Keywords:** Surgical oncology, Liver cancer

## Abstract

Aiming to improve the postoperative outcome of associating liver partition and portal vein ligation for staged hepatectomy (ALPPS), the effect of physical prehabilitation (PP) was investigated in experimental model. Male Wistar rats (n = 106) divided to PP and sedentary (S) groups underwent ALPPS. Changes in liver weight, Ki67 index and liver volume by magnetic resonance imaging (MRI) were evaluated. Liver function was assessed by laboratory parameters and ^99m^Tc-mebrofenin single-photon emission computed tomography (SPECT) hepatobiliary scintigraphy (HBS). Utilizing endotoxemia model mortality and septic parameters were investigated. Liver mass (*p* < 0.001), Ki67 index (*p* < 0.001) and MRI liver volume (*p* < 0.05) increased in the PP group compared to the S group. Both standard laboratory parameters (*p* < 0.001) and HBS (*p* < 0.05) showed enhanced liver function in the PP group compared to the S group. The vulnerability of animals improved in the PP group, as mortality decreased (*p* < 0.001), while septic laboratory parameters improved (*p* < 0.05) compared to the S group in the endotoxemia model. Our study demonstrated for the first time the beneficial role of PP on not only volumetric but also functional liver regeneration and postoperative vulnerability after ALLPS.

## Introduction

Associating liver partition and portal vein ligation for staged hepatectomy (ALLPS) is a two-stage hepatectomy offered to patients with inoperable tumors by standard procedures^1^. Its unique attribute is the capacity to induce robust and rapid regeneration of the future liver remnant (FLR), which could be in favor of advanced stage hepatic cancer patients, when extended resectability is needed^2^. Counterbalancing the remarkable advantages of the procedure, initially high morbidity and mortality rates were reported^3^. Although due to better patient selection and technical development these unfavorable aspects have improved since^2^, the patomechanism behind the high mortality and morbidity rates is still not completely elucidated.

Reciprocity between volumetric and functional regeneration of the FLR has been described following ALPPS, which results in a delayed functional recovery and undoubtedly contributes to the occurrence of postoperative complications^4,5^. In our previous studies we found that impaired mitochondrial function and biogenesis could be accounted for the delayed functional regeneration after ALPPS^6^. Seeking for methods to improve cell energy supply, we found that physical prehabilitation (PP) applied as preoperative exercise notably enhanced both mitochondrial biogenesis and function while also induced an even more potent regeneration following ALPPS^7^.

Therefore, we hypothesize that by stabilizing the cell-energy homeostasis PP could also improve liver function and the postoperative outcome following ALPPS. PP has been reported to enhance the function of various organs as enhanced respiratory and circulatory functions are associated with physical exercise^8,9^, which has been postulated to contribute to the overall recovery following liver surgery^10^. However, there is a paucity of data regarding the effects of exercise on liver function. To our knowledge, there is no literature data about the influence of PP on the postoperative outcome after ALPPS. Thus, in the current study we aimed to identify the effect of PP on liver regeneration, liver function and postoperative vulnerability following ALPPS.

## Results

### PP accelerates liver mass growth, cell proliferation and FLR volume (FLRV) increase after ALPPS

**Figure 1:**
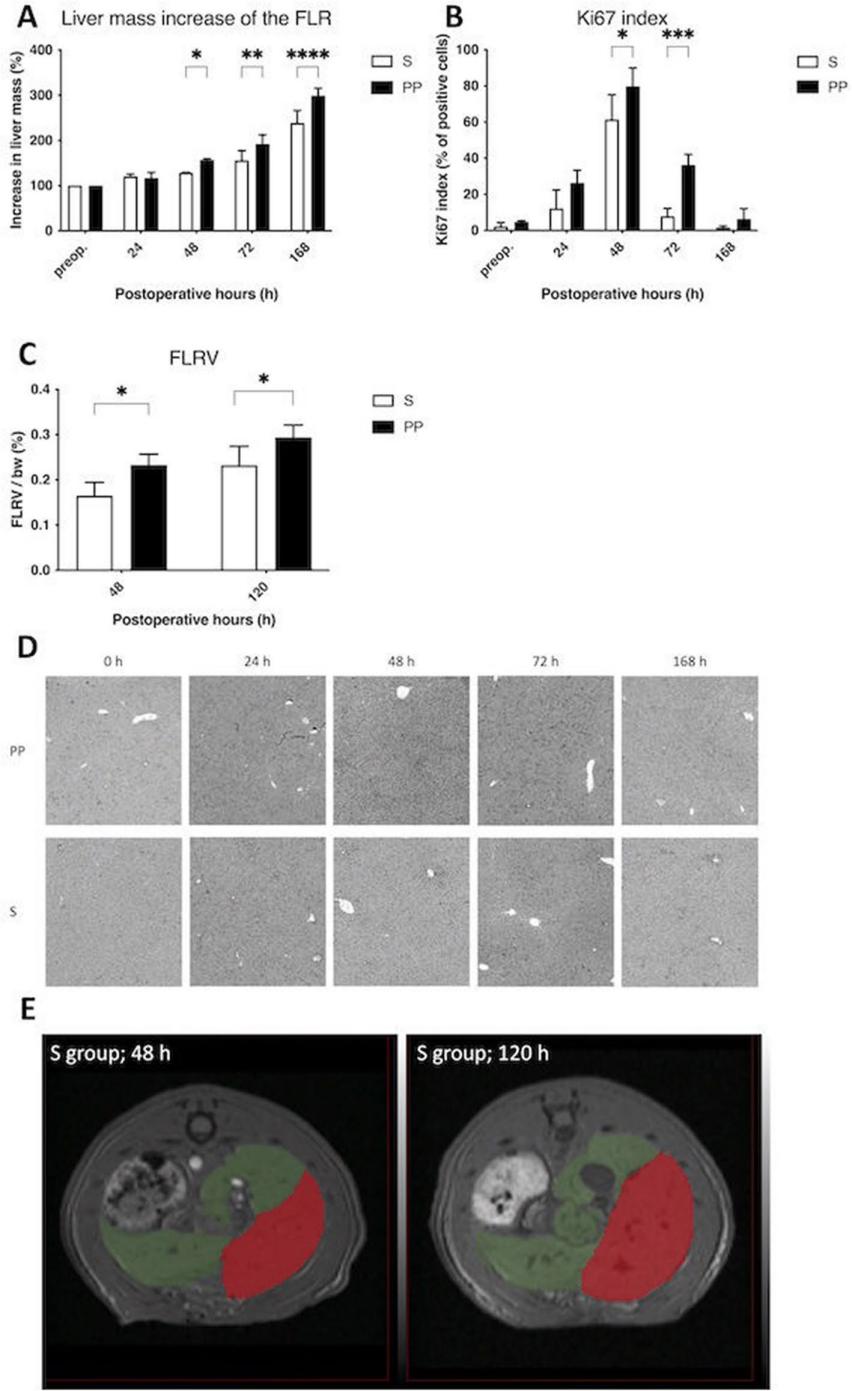
Liver regeneration of the future liver remnant (FLR). Increase in liver mass of the functional liver remnant (FLR) (**A**) and ki67 index (**B**, **D**) preoperatively (preop., 0 h), and at 24 h, 48 h, 72 h, and 168 h after associating liver partition and portal vein ligation for staged hepatectomy (ALPPS) (N = 6 per time point per group). FLR volume (FLRV) (**C**) at 48 h and 120 h after ALPPS (N = 5 per time point per group). Representative magnetic resonance imaging (MRI)-volumetry picture (**E**) of volume increase of FLR (red mark) and volume decrease of the ligated lobes (green mark) in the S group 120 h after ALPPS. *p < 0.050, **p < 0.0010,  *** p < 0.00010, **** p < 0.000010 physical prehabilitation (PP) versus sedentary (S).

ALPPS induced liver growth of the FLR both in the sedentary (S) (238.85 ± 27.31 percent) and PP (299.16 ± 16.38 percent) groups, however the increase in liver mass was more expressed in the PP group, resulting in a significant difference between the groups from 48 h until 168 h (Fig. 1A). Validating our model, no difference could be observed between the two groups regarding neither the preoperative total liver weight, nor the weight of the FLR (Supplemental Figure SF1). Cell proliferation in the FLR also increased in both groups from 24 to 72 h, reached the peak at 48 h and decreased to the baseline by the end of the experiment. Supporting the increase observed in liver mass, Ki67 index was also higher in the PP group at 48 and 72 h compared to the S group (Fig. 1B,D). Magnetic resonance imaging (MRI) liver volumetry also showed increased volume of FLR from 48 to 120 h in the S and PP group, whereby FLRV in the PP group was higher both at 48 and 120 h than in the S group (Fig. 1C,E).

### PP enhances laboratory parameters of liver function after ALPPS

**Figure 2:**
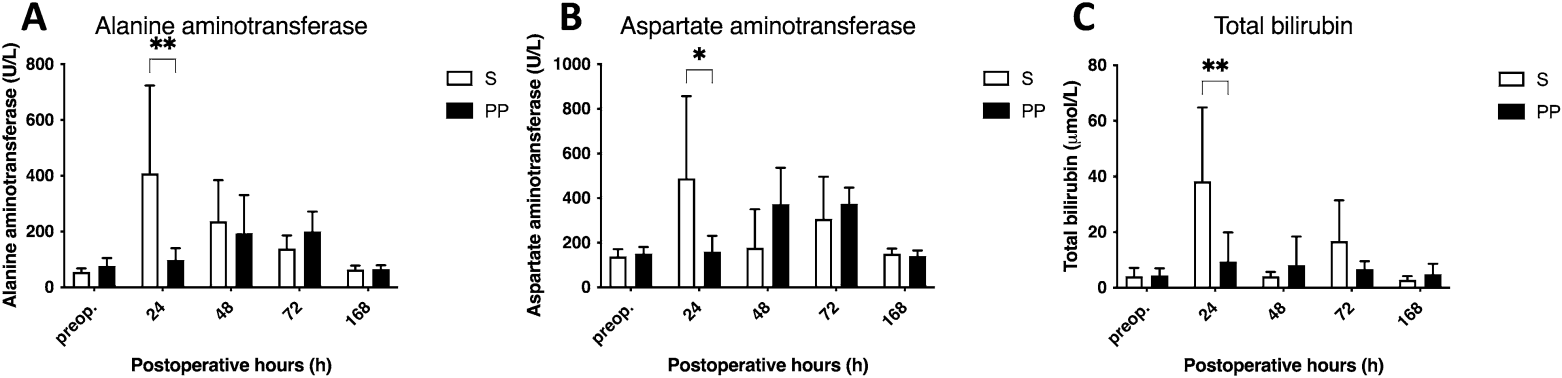
Changes in liver function laboratory parameters. Level of alanine aminotransferase (**A**), aspartate aminotransferase (**B**) and total bilirubin (**C**) preoperatively (preop., 0 h), and at 24 h, 48 h, 72 h, and 168 h after associating liver partition and portal vein ligation for staged hepatectomy (ALPPS) (N = 6 per time point per group). *p < 0.050, **p < 0.0010 physical prehabilitation (PP) versus sedentary (S).

The level of alanine-aminotransferase (ALT) increased significantly at 24 h in the S group and abated towards baseline level by the end of the experiment, while in the PP group the increase was less explicit. Therefore, ALT level was significantly lower in the PP group at 24 h (Fig. 2A). Aspartate-aminotransferase (AST) level changed by the same dynamic, as it sharply peaked in the S group at 24 h and remained lower in the PP group during the experiment resulting in a disparity between the groups at 24 h (Fig. 2B). Similarly, total bilirubin (tBil) level increased in the S group at 24 h and decreased by the end of the experiment, while there was no change in the PP group. Consequently, tBil level was significantly lower in the PP group compared to the S group at 24 h (Fig. 2C).

### PP ameliorates liver function measured by ^99m^Tc-mebrofenin hepatobiliary scintigraphy (HBS) after ALPPS

**Figure 3:**
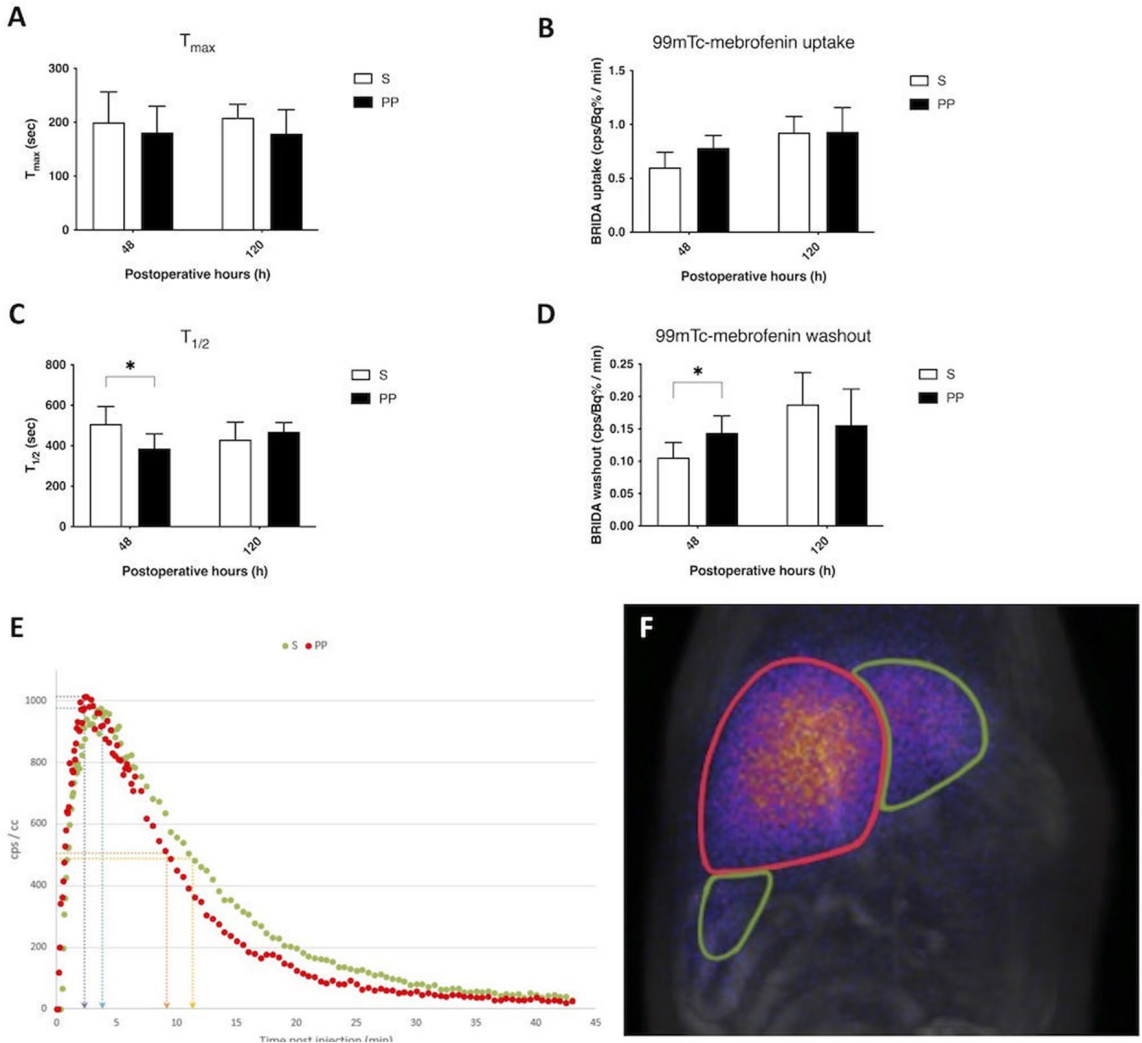
Changes in liver function measured by 99mTc-mebrofenin hepatobiliary scintigraphy (HBS). Time of maximum (T_max_) 99mTc-mebrofenin concentration (**A**), total 99mTc-mebrofenin uptake (**B**), tracer half-life (T_1/2_) 99mTc-mebrofenin concentration and total 99mTc-mebrofenin washout (**D**) preoperatively (preop., 0 h), and at 24 h, 48 h, 72 h, and 168 h after associating liver partition and portal vein ligation for staged hepatectomy (ALPPS) (N = 6 per time point per group). Representative curve of 99mTc-mebrofenin concentration in the change of time (**E**) with T_max_ of the PP group (purple arrow), T_max_ of the S group (blue arrow), T_1/2_ of the PP group (orange arrow) and T_1/2_ of the S group (yellow arrow). Representative figure of 99mTc-mebrofenin HBS 48 h after ALPPS in the PP group (**F**) showing a notable difference of 99mTc-mebrofenin uptake between the functional liver remnant (FLR) and the ligated lobes . *p < 0.050, **p < 0.0010 physical prehabilitation (PP) versus sedentary (S).

Time of maximum (T_max_) in the FLR, which characterizes the organic anion uptake capacity of the liver, did not show difference between the S and PP group (Fig. 3A,E,F). By the same token, there was no significant disparity in ^99m^Tc-mebrofenin uptake between the groups (Fig. 3B,E,F). However, tracer half-life (T_1/2_) in the FLR was significantly lower at 48 h in the PP group compared to the S group, indicating a more effective hepatic excretion after ALPPS in the PP group (Fig. 3C,E,F). Supporting this, ^99m^Tc-mebrofenin washout was significantly higher in the PP group compared to the S group at 48 h (Fig. 3D,E,F), too.

### PP deteriorates vulnerability in the lipopolysaccharide (LPS) endotoxemia model after ALPPS

**Figure 4:**
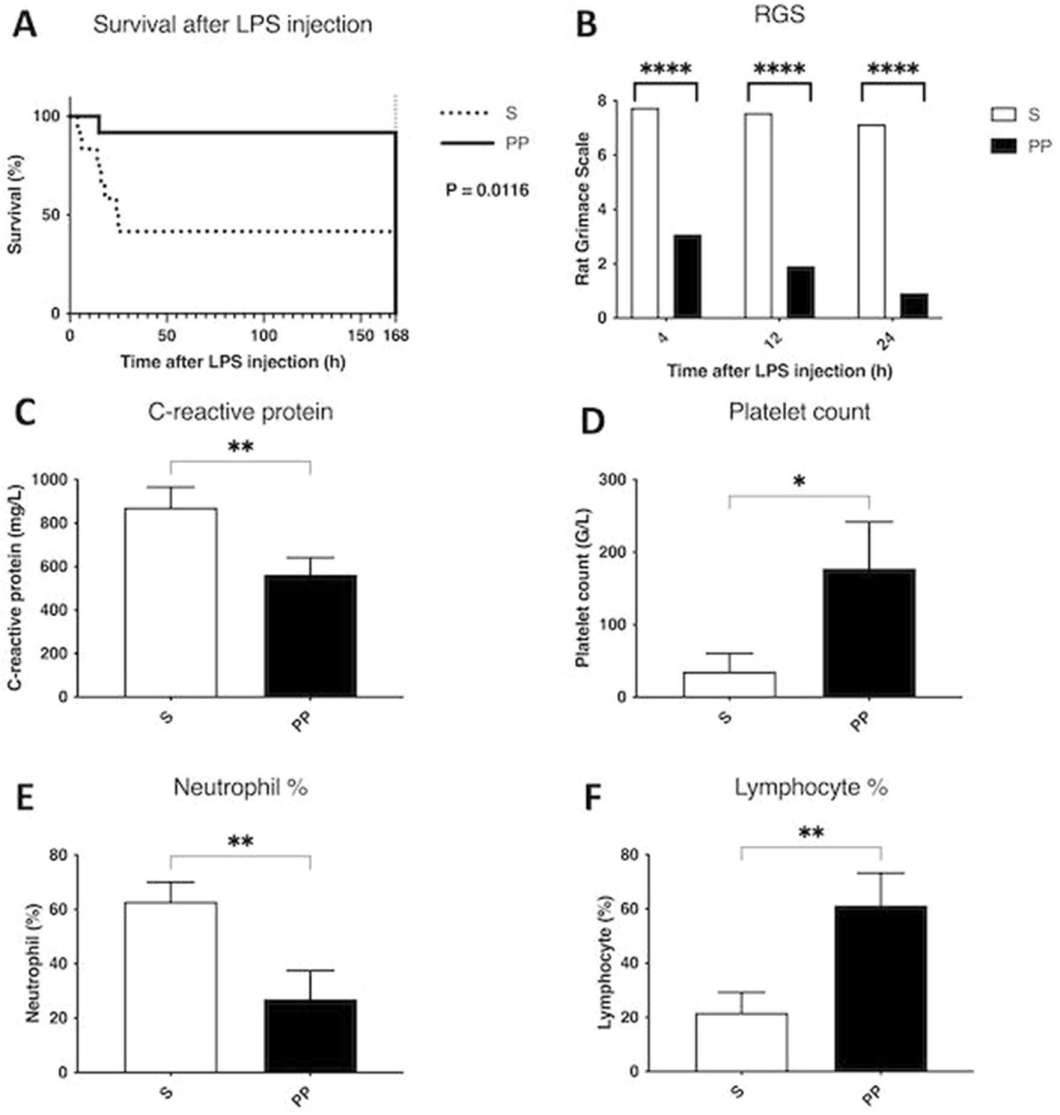
Changes in the vulnerability of the animals in lipopolysaccharide (LPS) endotoxemia model. Survival after LPS injection following associating liver partition and portal vein ligation for staged hepatectomy (ALPPS) monitored for 168 h (**A**). Rat Grimace Scale (RGS) monitored at 4 h, 12 h and 24 h after LPS injection utilized following associating liver partition and portal vein ligation for staged hepatectomy (ALPPS) (**B**). C-reactive protein (**C**), platelet count (**D**), neutrophil percent (%) (**E**) and lymphocyte % (**F**) monitored at 24 h after LPS injection utilized following associating liver partition and portal vein ligation for staged hepatectomy (ALPPS). *p < 0.050, **p < 0.0010, ****p < 0.00001 physical prehabilitation (PP) versus sedentary (S) (N = 6 per time point per group).

Vulnerability of the animals was assessed after the operation by inducing endotoxemia with LPS injection. A notable difference could be observed between the two groups, as the survival rate after LPS injection was 91.67 percent in the PP group, while only 41.67 percent in the S group, indicating an excessively increased stress-tolerance in the PP animals (Fig. 4A). Underpinning this, the Rat Grimace Scale (RGS) showed significantly lower values in the PP group compared to the S group, suggesting reduced pain in the PP animals after LPS injection (Fig. 4B). In line with the above, laboratory results also showed better stress-tolerance in the PP group, as the CRP level was higher (Fig. 4C), platelet count lower (Fig. 4D), neutrophil % higher (Fig. 4E) and lymphocyte % lower (Fig. 4F) in the S animals compared to the PP group, indicating markedly expressed inflammation, thrombocytopenia, neutrophilia and lymphocytopenia in the sedentary animals.

### PP improves body composition, which correlates with liver regeneration after ALPPS

**Figure 5:**
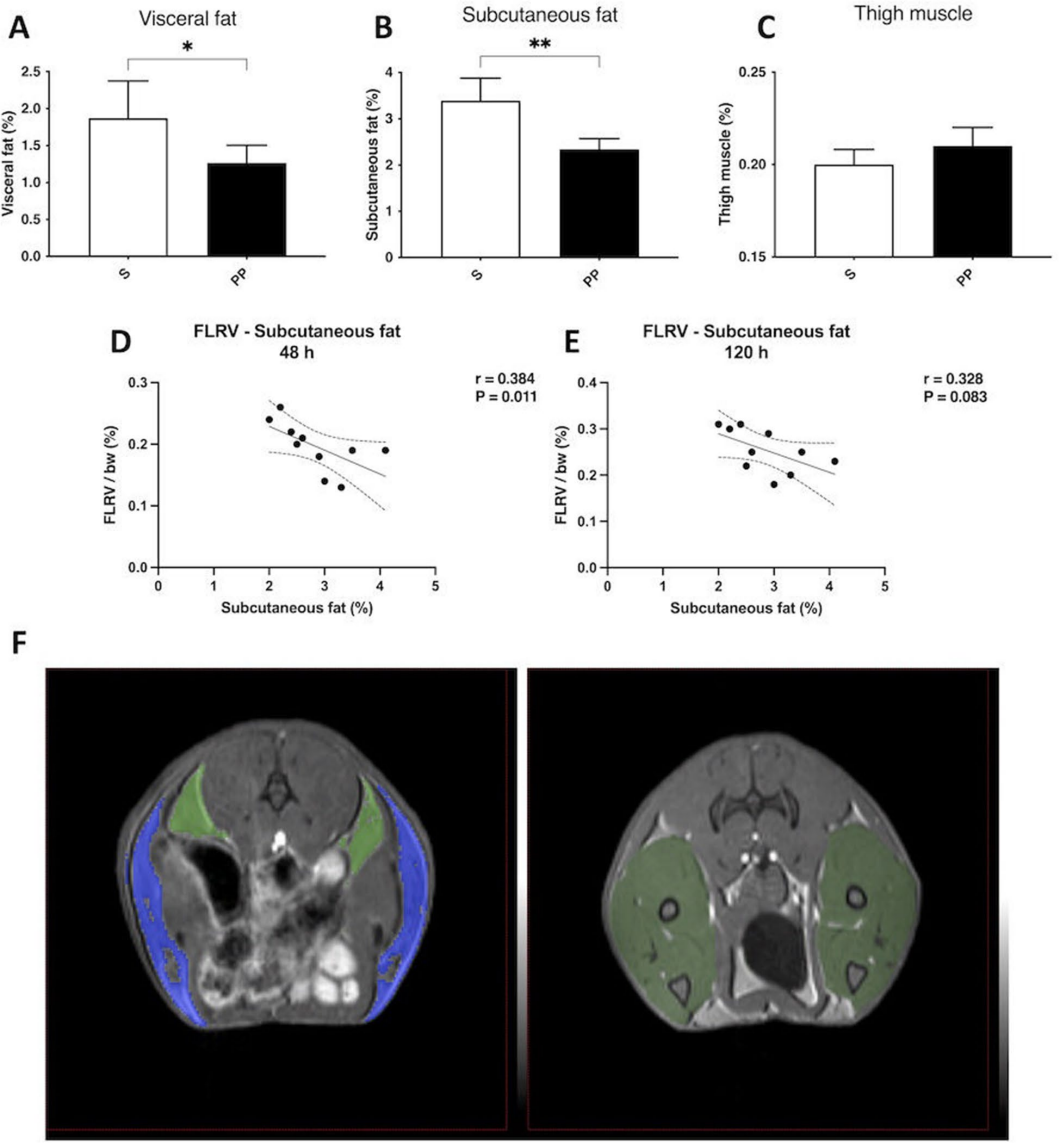
Preoperative magnetic resonance imaging (MRI)-volumetry of body composition and correlation between subcutaneous fat and future liver remnant volume (FLRV). Changes in visceral fat (**A**), subcutaneous fat (**B**) and thigh muscle (**C**) preoperatively. Linear regression analysis of correlation between subcutaneous fat and FLRV at 48 h (**D**) and 120 h (**E**) after associating liver partition and portal vein ligation for staged hepatectomy (ALPPS). Representative figure of preoperative MRI-volumetry (**F**) of the subcutaneous fat (blue), visceral fat (light green) and thigh muscle (dark green) in the PP group. *p < 0.050, **p < 0.0010 physical prehabilitation (PP) versus sedentary (S).

Confirming that exercise was properly implemented, the body fat composition of the animals in the PP group improved, as the percentage of both visceral (Fig. 5A,F) and subcutaneous (Fig. 5B,F) fat was reduced in the PP group compared to the S group on the preoperative MRI. There was no significant difference between the two group regarding muscle volume (Fig. 5C,F).

On univariable linear regression analysis, the subcutaneous fat percentage correlated with FLRV at 48 h, while only a tendentious correlation could be observed at 120 h (Fig. 5D-E). No correlations were found between either visceral fat percentage and FLRV, nor subcutaneous or visceral fat percentage and T_max_, T_1/2_, 99mTc-mebrofenin uptake and washout.

## Discussion

ALPPS is a variation of two-stage hepatectomy that has been advocated for FLR augmentation to enable safe resection of hepatic tumors, which were initially considered unresectable^1^. Compared to conventional two-stage hepatectomies, ALPPS accelerates robust liver hypertrophy and reduces the time between the two steps of the surgery^11^. Along with its beneficial effects, adverse events also occurred postoperatively, as in the beginning morbidity and mortality rates (40% and 9% respectively) were undeniably high^12^. Considering these disadvantageous aspects, the potential perks of ALPPS over conventional liver volume manipulation techniques could only be in favor of patients if the postoperative outcome becomes at least comparable to these procedures. In the last few years, a new concept called prehabilitation has seen the light in oncological surgery, which aims to improve patients’ bearing capacity and decrease the perioperative stress^13^. One entity of prehabilitation is the improvement of the physical state, which is extensively investigated in colorectal surgery and its positive impact on postoperative outcome has been also supported by our previous study, which recommends preoperatively 4 weeks of walking-breathing exercise^14^. As with hepato-pancreato-biliary surgery, unfortunately literature data are scarcer and more controversial^15–18^, and the physiological effects of preoperative exercise are still less known. In our previous study PP was proven to stabilize mitochondrial function following ALPPS^7^. Based on these, we hypothesized that PP could also enhance the otherwise lagging functional recovery of the liver after ALPPS and hence, improve postoperative outcomes.

To our knowledge, this is the first study investigating the influence of PP on the postoperative outcomes of ALPPS. Remarkably, our main findings showed that PP enhanced not only [1] liver regeneration, [2] but also improved postoperative liver function and [3] reduced postoperative vulnerability.

Adding to the essentially robust liver regeneration induced by ALPPS, PP accelerated an even more pronounced liver growth, with an increase in liver mass of more than 60% compared to the sedentary animals, which is line with our previous investigation^7^. Serving as a proof of concept and model reliability, both cell division in the FLR and the FLRV measured by MRI were notably higher in the animals receiving PP.

Unfortunately, the rapid volumetric regeneration is not necessarily followed by the functional regeneration of the liver after ALPPS, resulting in functionally immature liver^5,19^. Our previous investigation on ALPPS showed the energetic disbalance of the regenerating liver caused by mitochondrial dysfunction, which could certainly contribute to the lagging functional recovery^6^. In our further study, PP has been proven to greatly enhance mitochondrial function after ALPPS^7^, hence, we postulated that it could also boost the functional regeneration of the liver. Confirming our hypothesis, liver transaminase and bilirubin levels did not raise in the PP group, indicating the functional intactness of the liver, while a sharp peak could be observed in the sedentary animals at 24 h, which is also supported by previous findings on ALPPS^20^. Unfortunately, standard laboratory parameters are only informative on whole liver function, greatly limiting their applicability. For this reason, we have also investigated liver function with the nuclear imaging method ^99m^Tc-mebrofenin HBS, which could selectively measure the segmental function of the liver and allows the differentiation between hepatic uptake and excretion. As it was found to accurately measure FLR function after two-stage hepatectomy both in experimental and clinical reports, it is currently considered a highly relevant method for the assessment of hepatic function following ALPPS^5,21^. In accordance with laboratory parameters, ^99m^Tc-mebrofenin HBS showed enhanced FLR function in the PP animals compared to the sedentary animals. Strikingly, the change was notable only in hepatic excretion, while no difference could be detected between the groups when uptake was assessed. As multidrug resistance-associated protein (MRP) 2 and MRP3 play a central role in the excretion of ^99m^Tc-mebrofenin^22^, the altered function of these transporters could explain the improvement in the hepatic excretory function. Ventricular upregulation of MRP2 has been already revealed as an effect of exercise^23^. While further investigations about the influence of PP on liver transporters are needed for confirmation, this phenomenon is echoed in our results. Although literature data are available about the favorable aspects of prehabilitation after hepatectomy^15–17,24^, the present study is the first known demonstration of the effects of PP on both whole and regional liver function, moreover no previous study investigated these changes either after liver regenerative surgery, nor in healthy liver.

In the wake of experienced dramatic changes in hepatic functional regeneration during the first 24–48 h, which is the most vulnerable period following two-stage hepatectomies^25^, we embarked to further investigate whether PP has an impact on postoperative vulnerability. Utilizing an LPS-induced endotoxemia model after ALPPS, remarkable changes manifested in postoperative mortality. Survival was more than 90 percent in the PP group, whereas it dimidiated to close to 41 percent in the S group, demonstrating the improved stress-tolerance of the PP animals. Corroborating with this, sepsis-related laboratory parameters such as CRP, thrombocytopenia, neutrophilia and lymphocytopenia also showed improved values in the PP group.

Since our results are novum, there is a need to validate the physical prehabilitation model utilized in our experiments, therefore relevant data were benchmarked against reports in literature. Firstly, we observed notably decreased visceral and subcutaneous fat in the PP animals on preoperative MRI, which is utterly in accordance with previous findings^26,27^. Secondly, our model yielded the same results for the utilized exercise modality as described by others^26–28^. It has been already elucidated that while aerobic training improves body fat composition, it does not have an impact on muscle growth, as resistant training is the adequate modality to enhance muscle development^28^. Similarly, treadmill running used in our model as a classical form of aerobic training did not have an influence on muscles, corroborating with the evidence in literature.

Given the evidence of improved body fat composition in our model, we were eager to investigate whether a correlation occurs with volumetric and functional changes of the liver. Statistical analysis of our data shows an early supportive effect of decreased subcutaneous fat in liver regrowth. To our best knowledge, we have evidenced for the first time that the percentage of subcutaneous fat correlates with the volumetric growth of the liver in the early and most vulnerable phase of liver regeneration after ALPPS. Regarding functional regeneration no such correlation was found.

It must be acknowledged that animal models yield some limitation. The most important difference between the rat model and the clinical practice of ALPPS is set in the size and lobular structure of the liver. However, this being a standard model species in liver surgery, our rat model approach was carefully designed, as with the ligation of the right lateral, left part of the median, left lateral, and caudate lobes leading to the portal vein, 80 percent of the liver is deportalized and the transection is performed between the ischemic line of the right and left median lobe, which resembles well the clinical situation.

In conclusion, our study revealed the beneficial impact of physical prehabilitation on both volumetric and functional regeneration of the liver after ALPPS. Moreover, evidence was raised that preoperative exercise by enhancing stress-tolerance improves the postoperative outcomes following ALPPS. Based on these findings, the disadvantageous aspects of ALPPS could be mitigated with physical prehabilitation, therefore we propose the further clinical investigation of preoperative aerobic physical exercise protocols to improve patients’ safety after surgery.

## Methods

All experiments were performed in accordance with the relevant guideline of the directive 2010/63/EU of the European Parlament, and all methods were reported in accordance with the ARRIVE guidelines^28^. Experiments were approved by the Scientific Ethical Committee on Animal Experimentation of the National Department of Food Chain Safety (approval number: PEI/001/1732–6/2015). Male Wistar rats (Toxicoop, Hungary) weighing between 220–250 g were housed in 12-h day-night cycle, with temperature (20–22 °C) and humidity (40–70%) controlled environment. Ad libitum access to water and standard chow (Toxicoop, Hungary) was provided. Before the inclusion to the experiment, animals were acclimated for 7 days.

### Physical prehabilitation protocol and experimental design

PP protocol was established as previously described^6,7^. Animals in the PP group received PP one hour long 5 times/week for 5 weeks in form of treadmill running with 16 m/min. Animals in the sedentary group were housed in standard conditions for the same time interval (5 weeks) without receiving physical preconditioning. For detailed description of the protocol see Supplementary S1.

Experimental design consisted of three main groups (Supplemental Figure SF2). In *experiment 1* (SF 1A) 60 animals were included (n = 30 S, n = 30 PP). Animals were terminated preoperatively and at 24, 48, 72, 168 h after surgery. Thereafter, liver weight, immunohistochemical and clinical chemistry analyzes were performed. In *experiment 2* (SF 1B) 10 animals were included (n = 5 S, n = 5 PP). Liver volumetry analyzes MRI and liver function analyzes by 99mTc-mebrofenin hepatobiliary scintigraphy were carried out on the identical animals preoperatively and at 48 and 120 h after surgery. Animals were terminated at the end of the experiment. In *experiment 3* (SF 1C) 36 animals were included (n = 18 S, n = 18 PP). Animals received LPS injection 24 h following surgery (see 2.6.1.) and the surviving rats were terminated 48 and 192 h after surgery. Thereafter, total blood count test and C-reactive protein measurement was performed. Overall survival and rat grimace scale (RGS) were also determined.

### Surgical procedure

ALPPS operation was performed as previously described^6,7^. Briefly, following induction anesthesia (intraperitoneal injection of 75 mg/kg ketamine and 7.5 mg/kg xylazine in 1.5 mL saline solution) portal branches leading to the right lateral, left part of the median, left lateral, and caudate lobes were ligated. Alongside the transition line, which is situated between the left- and right part of the median lobe, transection was performed followed by careful electrocauterization of the liver wounds. Animals received antibiotic treatment (10 mg/kg body weight metronidazole intraperitoneally) and analgesia (1 mg/kg nalbuphine subcutaneously, repeated once 24 h post-operatively).

### Sample extraction

Following intraperitoneal injection of 75 mg/kg ketamine and 7.5 mg/kg xylazine in 1.5 mL saline solution the animals were terminated by exsanguination via cardiopuncture. Blood was collected in tripotassium (K3) ethylenediamin tetra-acetic acid (EDTA), citrate and lithium heparin (Vacutainer) tubes. Following the in toto extraction of the liver, approximately 150 mg tissue of the right median lobe was fixed in 4% buffered formaldehyde for histology.

### Assessment of morphologic alterations

#### Liver weight measurement

Wet weight of each lobe was determined by analytical scale (AG 245, Mettler-Toledo LLC, Columbus, OH; confidence: 0.01 mg/0.1 mg). Percentual increase of the FLR mass was defined by (lobe weight / body weight at the time of death) / (mean lobe weight at preoperative time point/ body weight at preoperative time point) × 100%.

#### Magnetic resonance imaging (MRI)-volumetry

In vivo liver lobe volumes were determined by 3 Tesla (3 T) MRI volumetry (nanoScan 3 T PET/MRI; Mediso Ltd., Budapest, Hungary) (for detailed description see S2). FLRV was expressed as a fraction of total liver volume and body weight.

#### Immunohistochemical analysis

Immunohistochemical analysis was performed as previously described^7^ (for detailed description see S3). The Ki67 index was calculated on the whole slide by the following formula: number of Ki67-positive cells / total number of cells.

### Assessment of liver function

#### Clinical chemistry

Plasma AST, ALT and tBil level was measured by Vetlabor Veterinary Clinic and Laboratory (Budapest, Hungary).

#### 99mTc-mebrofenin hepatobiliary scintigraphy (HBS)

Planar HBS (nanoScan SPECT/CT system, Mediso Ltd., Budapest, Hungary) was acquired after the injection of ^99m^Tc-mebrofenin (combination of Bromo-Biliaron ready-to-use radiopharmaceutical kit (Medi-Radiopharma Ltd., Budapest, Hungary), and ^99m^Tc isotope solution in physiological saline (Ultra-Technekow Technetium Generator, Mallinckrodt Medical, Petten, Netherlands)). For detailed methodology see S4. Characteristic parameters were computed from kinetic curves such as T_max_ and T_1/2_. The uptake of the FLR can be characterized by the midpoint of the ascending section of the curve, which fits well with a linear function. The difference in isotope concentration in the bound lobe and in blood was determined at two time points. This value is then normalized by the applied activity. The washout was determined with same method at the descendent section of the kinetic curve after the inflexion point of the isotope activity curve (Fig. 3E,F).

### Assessment of postoperative vulnerability

Postoperative vulnerability was assessed by creating a LPS induced endotoxemia model. LPS (O111:B4, L2630) was purchased from Sigma-Aldrich (St. Louis, Missouri, USA). Rats received intraperitoneal LPS injection (25 mg/body weight kg) 24 h after surgery.

#### Determination of survival and evaluation of animal well-being

Animal survival was determined by consecutive monitoring after LPS injection. Administration of RGS, which features specific facial action units increasing in intensity in response to post-procedural pain^29^, was performed 4, 12 and 24 h after LPS injection.

#### Total blood count test and quantification of C-reactive protein

C-reactive protein level, platelet count, neutrophil percentage (%) and lymphocyte % was determined by Vetlabor Veterinary Clinic and Laboratory (Budapest, Hungary).

### Assessment of body composition

In vivo fat and muscle volumes were determined by MRI volumetry (coronal T1-weighed gradient echo sequencing, 128 axial slices of 0.4 mm thickness) (nanoScan 3 T PET/MRI; Mediso Ltd., Budapest, Hungary) (Fig. 5F). In the first step the subcutaneous and visceral fat were manually roughly segmented using the appropriate size sphere. In the next step for clarification the subcutaneous and visceral fat layer were selected by choosing the density threshold between (10,000–18,000 voxel greyscale values in vivoQuant 1.22 software (inviCRO-Konica-Minolta Inc., Boston, US)). Total thigh volume was measured at the level of a single vertebral slice (lumbar L3). Measurements were performed in a semi-automated fashion with manual outlining of thigh muscle borders and setting the density.

### Statistical analysis

Data is expressed as mean ± standard deviation. Normality and homoscedasticity of the data was analyzed with diagnostic tests. Statistical analysis was performed using two-way ANOVA with Tukey’s test for post-hoc analysis, Student's t-test, Mantel-Cox log-rank test and univariable linear regression. For analysis of non-parametric data Mann–Whitney-U test was performed. A P-value ≤ 0.05 was considered statistically significant. Calculations and visualisation were performed with GraphPad Prism 9 (GraphPad Software Inc., La Jolla).

### Ethics declaration

All experiments are in compliance with the directive 2010/63/EU of the European Parlament, were reported in accordance with the ARRIVE criteria (11) and were approved by the Scientific Ethical Committee on Animal Experimentation of the National Department of Food Chain Safety (approval number: PEI/001/1732–6/2015).

## Supplementary Information

Supplementary Information.

## Data Availability

The data presented in this study are available upon request to the corresponding author.
