# Supplementary material for: Physical prehabilitation improves the postoperative outcome of associating liver partition and portal vein ligation for staged hepatectomy in experimental model
Source: Sci Rep. 2022 Nov 14;12:19441. doi: 10.1038/s41598-022-23744-2 (PMC9663729; doi:10.1038/s41598-022-23744-2)
Supplement: Supplementary file 1 — Supplementary Information. [file 41598_2022_23744_MOESM1_ESM.docx]

**Physical prehabilitation improves the postoperative outcome of**

**associating liver partition and portal vein ligation for staged hepatectomy**

**in experimental model**

Noemi Daradics ^1^, Klara Levay ^1^, Ildiko Horvath ^2^, Noemi Kovacs ^3^, Domokos Mathe ^2,3^, Krisztian Szigeti ^2^, Attila Szijarto ^1#^, Andras Fulop ^1#^

1 – Semmelweis University; Department of Surgery, Transplantation and Gastroenterology; Hepato-Pancreatico-Biliary Surgical Research Center; Budapest, Hungary

2 – Semmelweis University; Department of Biophysics and Radiation Biology, Budapest, Hungary

3 – Hungarian Centre of Excellence for Molecular Medicine, In Vivo Imaging Core Facility, Szeged, Hungary

# - The authors contributed equally to the article.

**Corresponding author:**

name: Noemi Daradics

email: daradics.noemi@phd.semmelweis.hu

address: Ulloi Street 78, Budapest, H-1082

phone: +36-20-670-1424

Supplementary Figure (SF) 1: Preoperative liver weight measurement


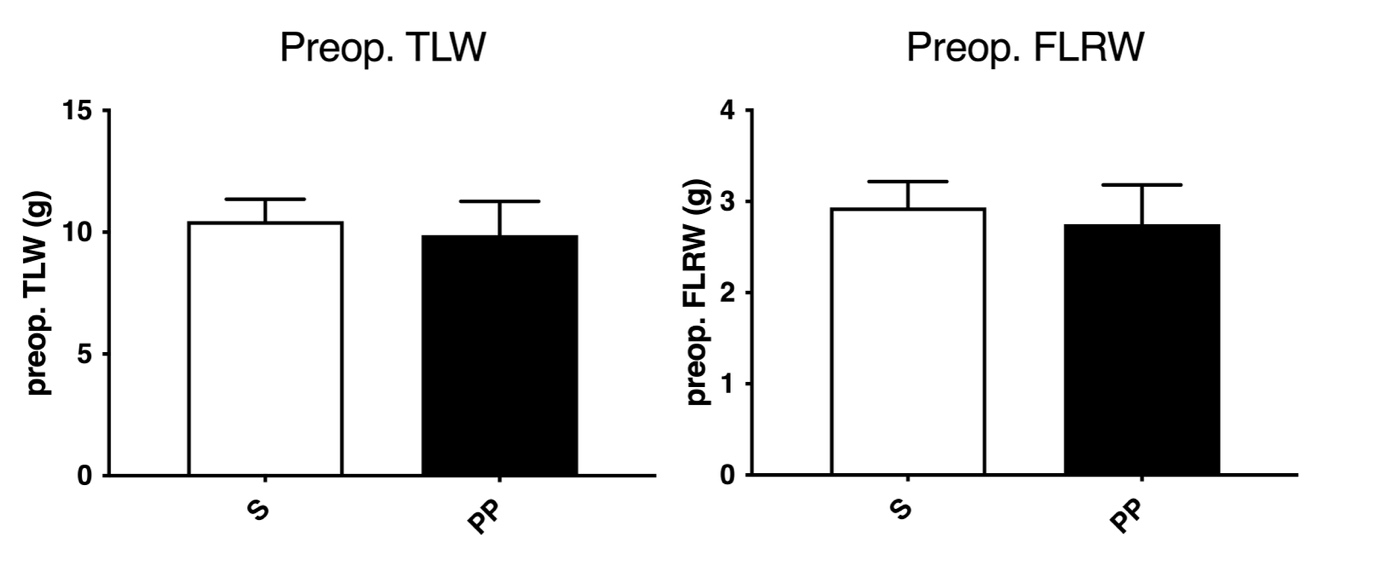


***SF 1. Preoperative liver weight measurement.*** The preoperative total liver weight (TLW) and future liver remnant weight (FLRW) in the sedentary (S) and physical prehabilitation (PP) groups following 5 weeks of standard conditions or physical prehabilitation respectively. (N = 6 per time point per group). * P < 0.050, ** P < 0.0010 physical prehabilitation (PP) versus sedentary (S).

Supplementary Figure (SF) 2: Experimental design


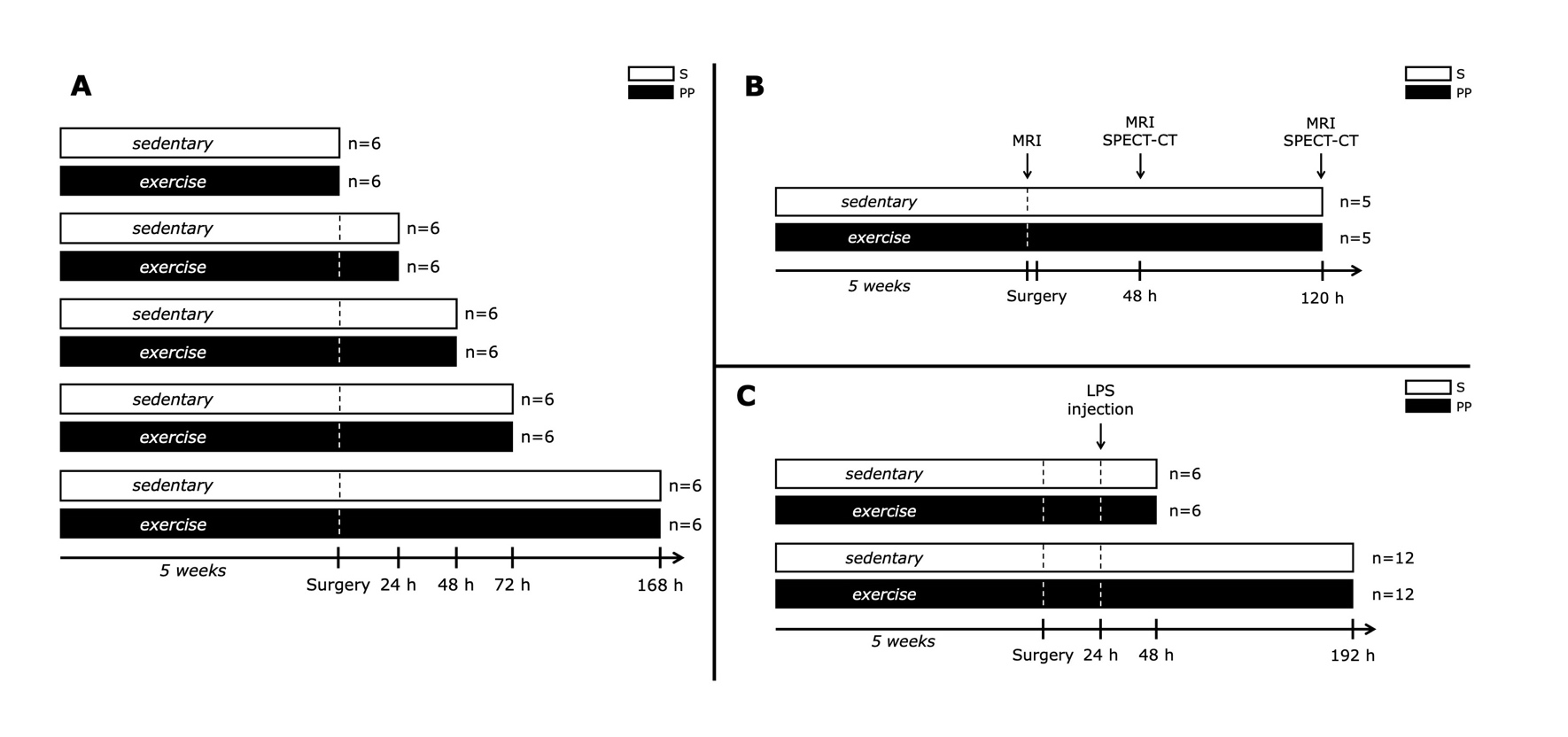


***SF 2. Experimental design.*** E*xperiment 1* (SF 1A) included 60 animals (n=30 S, n=30 PP). Time points for termination: preoperatively and at 24, 48, 72, 168 h after surgery. Analyzes performed: liver weight, immunohistochemical and clinical chemistry. E*xperiment 2* (SF 1B) included 10 animals (n=5 S, n=5 PP). Time point for termination: at the end of the experiment. Analyzes performed: liver volumetry by magnetic resonance imaging (MRI) and liver function by 99mTc-mebrofenin hepatobiliary scintigraphy at 48 and 120 h after surgery. *Experiment 3* (SF 1C) included 36 animals (n=18 S, n=18 PP). Animals received LPS injection 24 h following surgery (see 2.6.1.) and the surviving rats were terminated 48 and 192 h after surgery. Time points for termination: at the end of the experiment. Analyzes performed: total blood count test and C-reactive protein measurement. Overall survival and rat grimace scale (RGS) were also determined.

**Supplementary S1. Physical prehabilitation protocol**

Animals in the PP group received PP one hour long 5 times/week for 5 weeks in form of treadmill running with 16 m/min. The maximum running capacity of the rats – the maximum tolerable speed for 60 minutes without the signs of fatigue – was determined in a preliminary study (20 m/min). During the first week of exercise rats were accustomed to running by 8 m/min speed, which gradually increased each day by 1.6 m/min reaching 80% of the maximum tolerable running speed (16m/min) by the end of the first week. In the following 4 weeks rats continued the exercise with 16 m/min running speed. Animals in the sedentary group (S) were housed in standard conditions for the same time interval (5 weeks) without receiving physical preconditioning.

**Supplementary S2. Magnetic resonance imaging (MRI)-volumetry**

In vivo liver lobe volumes were determined by 3 Tesla (3T) magnetic resonance imaging (MRI) volumetry (coronal T1-weighed gradient echo sequencing, 128 axial slices of 0.4 mm thickness) (nanoScan 3T PET/MRI; Mediso Ltd., Budapest, Hungary). Manual delineation using vivoQuant 1.22 software (inviCRO-Konica-Minolta Inc., Boston, US) of the ligated lobes (LL; right lateral, left part of the median, left lateral, and caudate) and the non-ligated right median lobe, which is equal to the FLR, on each axial slice and three-dimensional reconstruction in a 160x160 matrix was performed.

**Supplementary S3. Immunohistochemical analysis**

4-µm thick sections were cut from the formalin fixed paraffin embedded liver tissue specimens. Afterwise sections were deparaffinized in xylene (2 × 10 min) and rehydrated in graded alcohol series. Antigen retrieval was accomplished at pH = 6.0 (S2031, Agilent, Santa Clara, CA). Ki67 immunohistochemistry was performed using anti-Ki67 antibodies (ab16667, Abcam, Cambridge, UK) according to the manufacturer’s instructions and was counterstained with hematoxylin. The histological slides were scanned using Pannoramic P1000 slide scanner system (3DHistech, Budapest, Hungary). Captured images were analyzed by QuPath software (12). The Ki67 index was calculated on the whole slide by the following formula: number of Ki67-positive cells / total number of cells.

**Supplementary S4. 99mTc-mebrofenin hepatobiliary scintigraphy (HBS)**

^99m^Tc-mebrofenin (combination of Bromo-Biliaron ready-to-use radiopharmaceutical kit (Medi-Radiopharma Ltd., Budapest, Hungary), and ^99m^Tc isotope solution in physiological saline (Ultra-Technekow Technetium Generator, Mallinckrodt Medical, Petten, Netherlands)), was injected in 140 MBq dosage in 0.13 ml saline into the tail vein. Thereafter, planar HBS (nanoScan SPECT/CT system, Mediso Ltd., Budapest, Hungary) was acquired from four angles in a resolution of 256 x 256 using Ultrahigh Resolution (UHR) parallel septal collimator (Mediso Ltd, Budapest, Hungary). A dynamic protocol of three different phases was used including 12/6/2 frames per minutes for 0.2/2.5/7.0 minutes, respectively, to monitor the rapid uptake and the canalicular elimination of the tracer. Recordings were evaluated with manual allocation of elliptic regions of interest (ROI) to the anteroposterior projection corresponding to the blood pool, as well as the FLR using the Fusion software (Mediso Ltd. Budapest, Hungary).
